# Influence of renal function and demographic data on intrarenal Doppler ultrasonography

**DOI:** 10.1371/journal.pone.0221244

**Published:** 2019-08-27

**Authors:** Michiaki Abe, Tetsuya Akaishi, Takashi Miki, Mika Miki, Yasuharu Funamizu, Kaori Araya, Kota Ishizawa, Shin Takayama, Kei Takase, Takaaki Abe, Tadashi Ishii, Sadayoshi Ito

**Affiliations:** 1 Department of Education and Support for Regional Medicine, Tohoku University Hospital, Sendai, Japan; 2 Department of Nephrology, Endocrinology and Vascular Medicine, Tohoku University Hospital, Sendai, Japan; 3 Clinical Physiology Center, Tohoku University Hospital, Sendai, Japan; 4 Diagnostic Radiology, Tohoku University Hospital, Sendai, Japan; University of Mississippi Medical Center, UNITED STATES

## Abstract

Intrarenal Doppler ultrasonography is a non-invasive method to evaluate the renal blood flow in patients with renal arterial stenosis as well as chronic kidney diseases (CKD). Until recently, the relationship between ultrasonography findings and CKD stage has not been fully understood. Overall, 162 patients with CKD without apparent renal arterial stenosis were included in this study, and the pulsed-wave Doppler ultrasonography findings were evaluated in terms of the following parameters: peak systolic velocity (PSV), end-diastolic velocity (EDV), and resistive index (RI) at the renal arterial trunk, hilum, segmental, and interlobar regions. Age showed a significant negative correlation with the estimated glomerular filtration rate (eGFR), kidney size, and aortic PSV. Additionally, age showed a significant positive correlation with RI in all 4 regions. The eGFR showed a positive correlation with the aortic PSV and kidney size, but a negative correlation with RI. Both age and eGFR were found to be independently associated with aortic blood flow. On the intrarenal ultrasound, EDV and RI showed stronger correlations with eGFR than PSV, suggesting that the former indices would be better markers of renal function. In particular, the interlobar EDV was found to be the best index that reflects renal function. Although the RI is also a good marker of renal function, it is confounded by age; thus, its utility would be weaker than that of the EDV. In conclusion, intrarenal pulsed-wave Doppler ultrasonography is a useful tool to estimate and evaluate the renal function; the interlobar EDV may be the best index to estimate the effective perfusion and filtration of the kidneys.

## Introduction

Doppler ultrasonography is a non-invasive method to evaluate blood flow in organs. In patients with chronic kidney disease (CKD) and renal arterial diseases, particularly renal arterial stenosis, evaluations of blood flow with pulsed-wave Doppler ultrasonography in the aorta, renal artery, and intrarenal small-to-middle sized arteries have been attempted previously [1,2]. Presently, the most commonly used marker of renal function is the serum creatinine level; but it is affected by age, sex, muscle mass, exercise, nutritional status, and hydration status. As a result, although it is a useful marker of renal function, serum creatinine is not a highly sensitive biomarker of renal hemodynamic parameters such as intrarenal blood flow. If clinicians and ultrasonographers can correctly perform and interpret a renal Doppler ultra-sonogram, it would be a more sensitive test for renal hemodynamics than the assessment of the serum creatinine level and can be quickly performed at every hospital visit.

Based on the wave pattern of arterial blood flow in each region on renal Doppler ultrasonography, the peak systolic velocity (PSV) and the end-diastolic velocity (EDV) of arterial flow can be evaluated. The level of vascular resistance, also known as the resistive index (RI), can be estimated from the PSV and EDV [3,4]. Until recently, the RI has been regarded as the most sensitive and reliable marker of disturbed intrarenal hemodynamics [5–7]. An increased RI has been known to indicate interstitial fibrosis and arteriosclerosis, which are eventually followed by a decline in renal function [8–11]. However, only a few reports have measured and compared PSV, EDV, and RI simultaneously to determine the most appropriate marker among these ultrasonic indices along with their clinical significance in patients with CKD [12]. Moreover, PSV and EDV have been previously considered to be useful for evaluating potential renal artery trunk stenosis, but their relationships with the intrarenal hemodynamics have not been enough evaluated. A comprehensive analysis with demographic variables including age is needed to correctly interpret the ultrasonic data.

This is the first study to comprehensively collect data regarding all ultrasonic indices (*i*.*e*. EDV, PSV, RI) from the aorta, trunk of the renal artery, renal hilum, segmental region, and interlobar regions in a sufficient number of CKD patients. Additionally, demographic and blood test data were analyzed to identify the covariates that can affect the correct interpretation of the acquired ultrasonic data. Based on the analyses, the most appropriate anatomical position and ultrasonic indices for assessment of CKD patients were determined.

## Materials and methods

### Ethical statements

This study was approved by the Institutional Review Board (IRB) of the Tohoku University Hospital (IRB No. 2015-1-765) and was performed in compliance with the Helsinki Declaration of 1964 and later versions. The IRB allowed a waiver of informed consent, because this was a retrospective observational study and was performed with non-invasive ultrasonography as part of the necessary medical practice.

### Subject enrollment

Blood and renal pulsed-wave Doppler ultrasonography data were collected from 204 patients with suspected CKD from 2007 to 2017. All the patients had undergone a renal Doppler ultrasonography to exclude renal arterial stenosis as part of the necessary diagnostic process, along with blood tests for plasma renin activity and plasma angiotensin II levels. Forty two patients were excluded due to apparent renal arterial stenosis, solitary kidney, or artificial dialysis to obtain reliable standard values. The globally recognized criterion to judge the presence of renal arterial stenosis were PSV >180 cm/sec and PSV renal/aortic ratio (RAR) >3.5 [13,14]. A total of 162 patients with CKD were enrolled and their renal Doppler ultrasonography data were analyzed.

### Collected data

Demographic data including sex, age at Doppler ultrasonography, body-mass index (BMI), and CKD stage were collected. Additionally, the history of hypertension, diabetes mellitus, dyslipidemia, and ischemic heart disease was included. Data from blood tests including serum creatinine level and estimated glomerular filtration rate (eGFR) were also collected. The value of eGFR was calculated using the following equation [15].

$$eGFR\;[ml/min/1.73m^{2}]\;(male)=194\times {Cr}^{-1.094}\times {Age}^{-0.287}$$

$$eGFR\;[ml/min/1.73m^{2}]\;(female)=eGFR\;(male)\times 0.739$$

The data of renal pulsed-wave Doppler ultrasonography were also comprehensively recorded for both kidneys, using a Doppler angle of ≤ 60 degree. The PSV, EDV, and RI were measured in the following 4 regions: trunk of the renal artery, hilum (renal pedicles), the segmental, and interlobar regions [16]. The PSV was also measured in the aorta (aortic PSV). These ultrasonic measurements were performed ≥ 3 times in each position and the averages were used as the measured values for the analyses. The RI was manually calculated from the measured values of the PSV and the EDV using the following equation [5].

$$RI=\frac{PSV-EDV}{PSV}$$

The ultrasonic data were measured for both kidneys and the average of both sides were used for the analyses. Acceleration time and pulsatility index were not measured in this study. The collected dataset from the enrolled subjects in this study is shown in **S1 Table**.

### Statistics and software

The correlation between 2 variables was evaluated using the Pearson’s correlation coefficient (*R*) and coefficient of determination (*R*^2^), followed by a test of no correlation. The correlation between 3 variables was evaluated by calculating the partial correlation coefficients. A comparison of multiple ultrasonic variables as markers of renal function was performed by a multiple linear regression analysis after setting eGFR as the objective variable. Before interpreting the results of the multiple regression analyses, the risk for multicollinearity was assessed using the variance inflation factor (VIF) for each explanatory variable.

Statistical analyses in this study were conducted using SPSS Statistics Base 22 software (IBM, Armonk, NY, USA), JMP Pro 14 (SAS Institute Inc., Cary, NC, USA) and MATLAB R2015a (MathWorks, Natick, MA, USA).

## Results

### Clinical information and distribution of study variables

The demographic and blood test data of the enrolled 162 patients with CKD are listed in **Table 1**. The eGFR of male patients (60.4±26.7) was slightly lower than that for female patients (70.8±30.8) (p = 0.0242, Student’s t-test). The ultrasonography indices in all 4 regions in both kidneys with the kidney sizes are listed in **Table 2**.

**Table 1:** Demographic data of 162 patients.

**Number (Male : Female)**	162 (97 : 65)
**Age at ultrasonography**	55.9±15.7
**SBP / DBP [mmHg]**	140.4±21.3 / 82.1±3.7
**BMI**	23.9±3.7
**Serum creatinine [mg/dl]**	1.20±1.11
**eGFR [ml/min/1.73 m**^**2**^**]**	64.5±28.8
**CKD stage (1 / 2 / 3A / 3B / 4 / 5)**	29 / 66 / 25 / 23 / 10 / 9
**Hypertension**	n = 147 (90.7%)
**Diabetes mellitus**	n = 35 (21.6%)
**Dyslipidemia**	n = 40 (24.7%)
**Ischemic heart disease**	n = 9 (5.6%)

The values and ranges are mean ± standard deviation for each variable. Abbreviations: BMI, body-mass index; CKD, chronic kidney disease; DBP, diastolic blood pressure; eGFR, estimated glomerular filtration rate; SBP, systolic blood pressure.

**Table 2:** Laterality and averages of measured ultrasonic indices.

		Right kidney	Left kidney	p	Average
**Kidney size [mm]**		101.7±9.9	103.9±10.3	0.618	103.0±9.4
**Trunk**	**PSV [cm/sec]**	93.3±29.0	83.1±26.3	0.0011	88.2±23.7
	**EDV [cm/sec]**	28.0±11.3	24.1±9.8	0.0011	26.0±9.3
	**RI**	0.692±0.091	0.704±0.091	0.268	0.699±0.086
**Hilum**	**PSV [cm/sec]**	68.6±21.8	66.2±20.6	0.336	67.8±19.7
	**EDV [cm/sec]**	22.7±20.3	20.4±8.4	0.206	21.8±12.8
	**RI**	0.688±0.089	0.691±0.089	0.789	0.689±0.083
**Segmental**	**PSV [cm/sec]**	43.6±10.7	44.1±12.0	0.729	43.8±10.5
	**EDV [cm/sec]**	16.1±17.5	15.3±9.5	0.608	15.7±10.7
	**RI**	0.660±0.091	0.664±0.090	0.747	0.662±0.087
**Interlobar**	**PSV [cm/sec]**	29.7±8.8	29.4±8.5	0.762	29.5±8.2
	**EDV [cm/sec]**	9.9±3.5	9.9±3.5	0.863	9.9±3.2
	**RI**	0.657±0.091	0.656±0.094	0.942	0.656±0.088

The velocity of blood flow in the trunk was significantly faster on the right side, but other indices did not show any laterality. The p-values are results from the Student’s t-test. Abbreviations: PSV, peak systolic velocity; RI, resistive index.

### Aortic and renal hemodynamics by age

**Fig 1:**
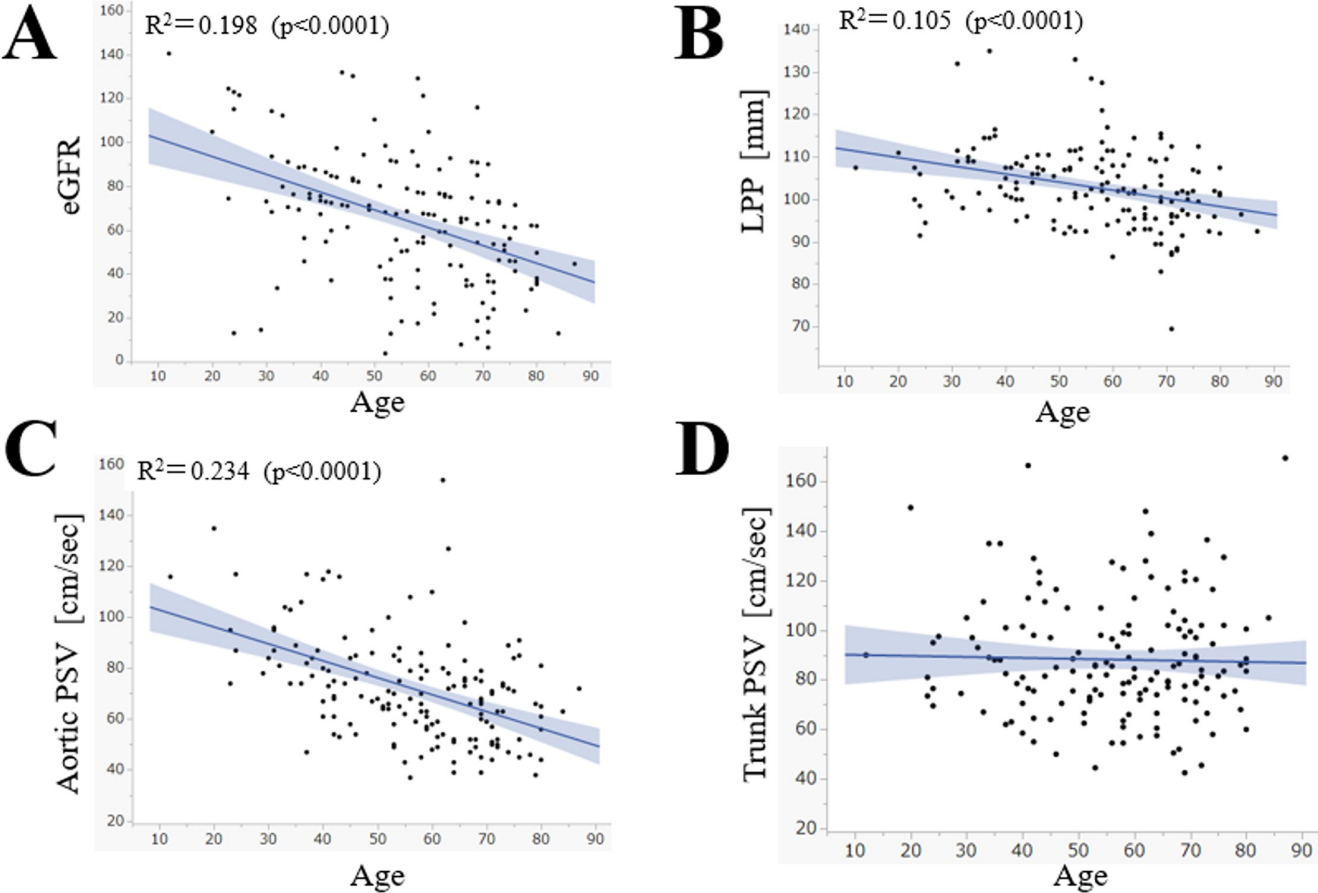
Scatter plots of age and other variables. Plot graphs A, B, C for eGFR, kidney size, and aortic blood flow velocity, respectively, show significant negative correlations with age. Plot graph D for the PSV in the trunk of the renal artery was not affected by age. The grey shaded ranges show the 95% confidence interval of the regression lines. Abbreviations: eGFR, estimated glomerular filtration rate: LPP, kidney length pole-to-pole; PSV, peak systolic velocity.

The scatter plots of age and other variables (*i*.*e*. eGFR, kidney size, aortic PSV, trunk PSV) are shown in **Fig 1**. The eGFR, kidney length pole-to-pole (LPP), and aortic PSV showed weak to moderate negative correlation with age, but not with the trunk PSV.

**Fig 2:**
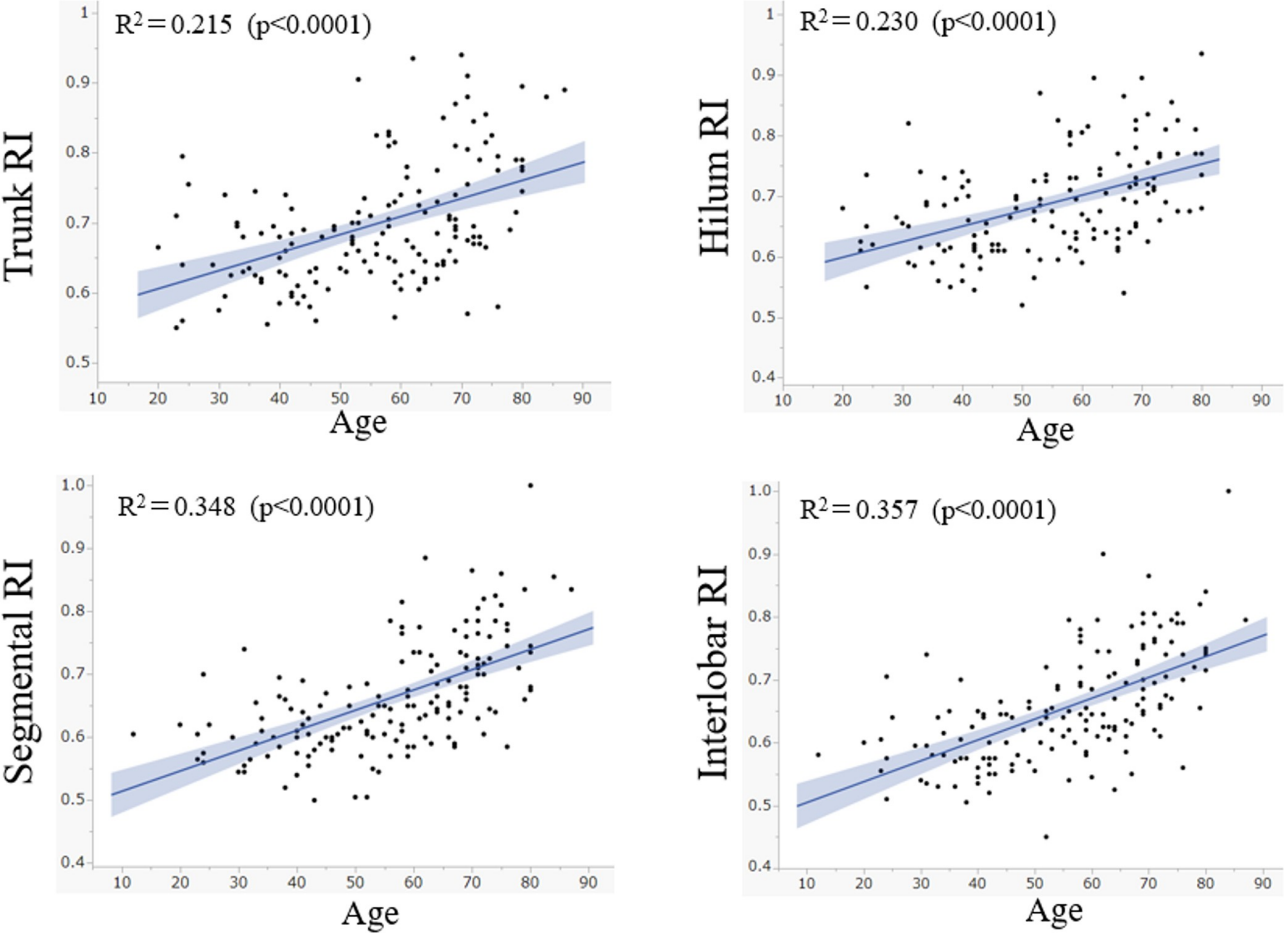
Scatter plots of age and resistive index in each region as assessed with Doppler ultrasonography. Resistive indices in all 4 regions show significant positive correlations with age. The grey shaded ranges show the 95% confidence interval of the regression lines. Although linear regression was attempted, non-linear regression may be more appropriate for these distributions.

The scatter plots of age and RI for all 4 regions are shown in **Fig 2**. The values for all 4 regions showed moderate correlations with age. The correlation was suggested to be stronger on the distal side (interlobar) and weaker on the proximal side (trunk), but the difference did not reach statistical significance (p = 0.099).

### Aortic PSV and kidney size by CKD stages

**Fig 3:**
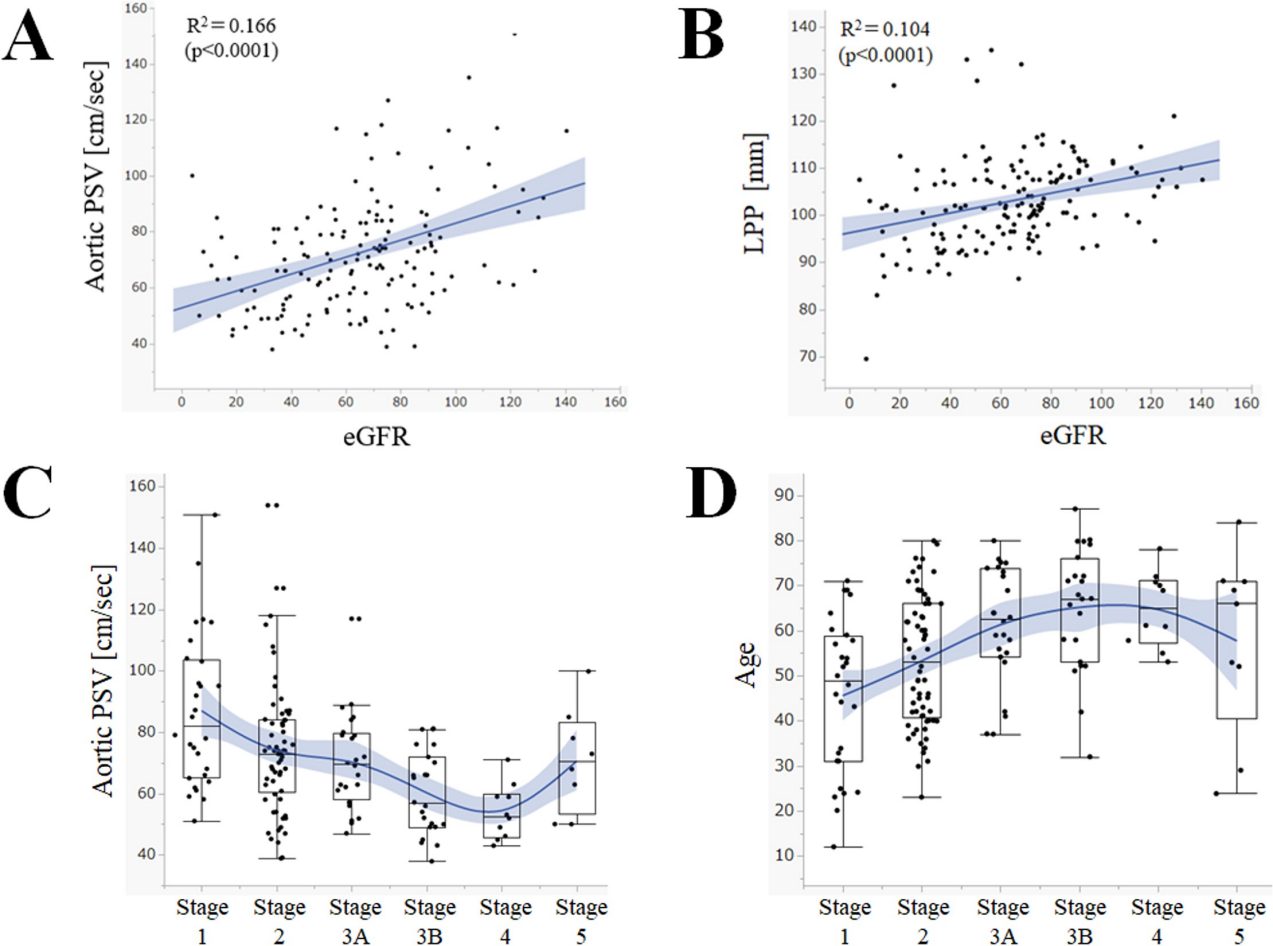
Scatter plots and box-whisker plots with renal function and other variables. Plot graphs A and B of the aortic PSV and the kidney size show positive correlations with eGFR. The grey shaded ranges show the 95% confidence interval of the regression lines. Graphs C and D show that aortic PSV and age were significantly affected by the CKD stages. The grey shaded ranges show the 95% confidence interval of the estimated averages for each CKD stage. Abbreviations: eGFR, estimated glomerular filtration rate; LPP, kidney length pole-to-pole; PSV, peak systolic velocity.

The scatter plots of the eGFR and other variables (*i*.*e*. aortic PSV, LPP) are shown in **Fig 3A and 3B**, with both showing weak to moderate correlations with eGFR. The aortic PSV and age in each CKD stage are shown in **Fig 3C and 3D**. It can be suggested that age was a significant covariate between the aortic PSV and renal function.

**Fig 4:**
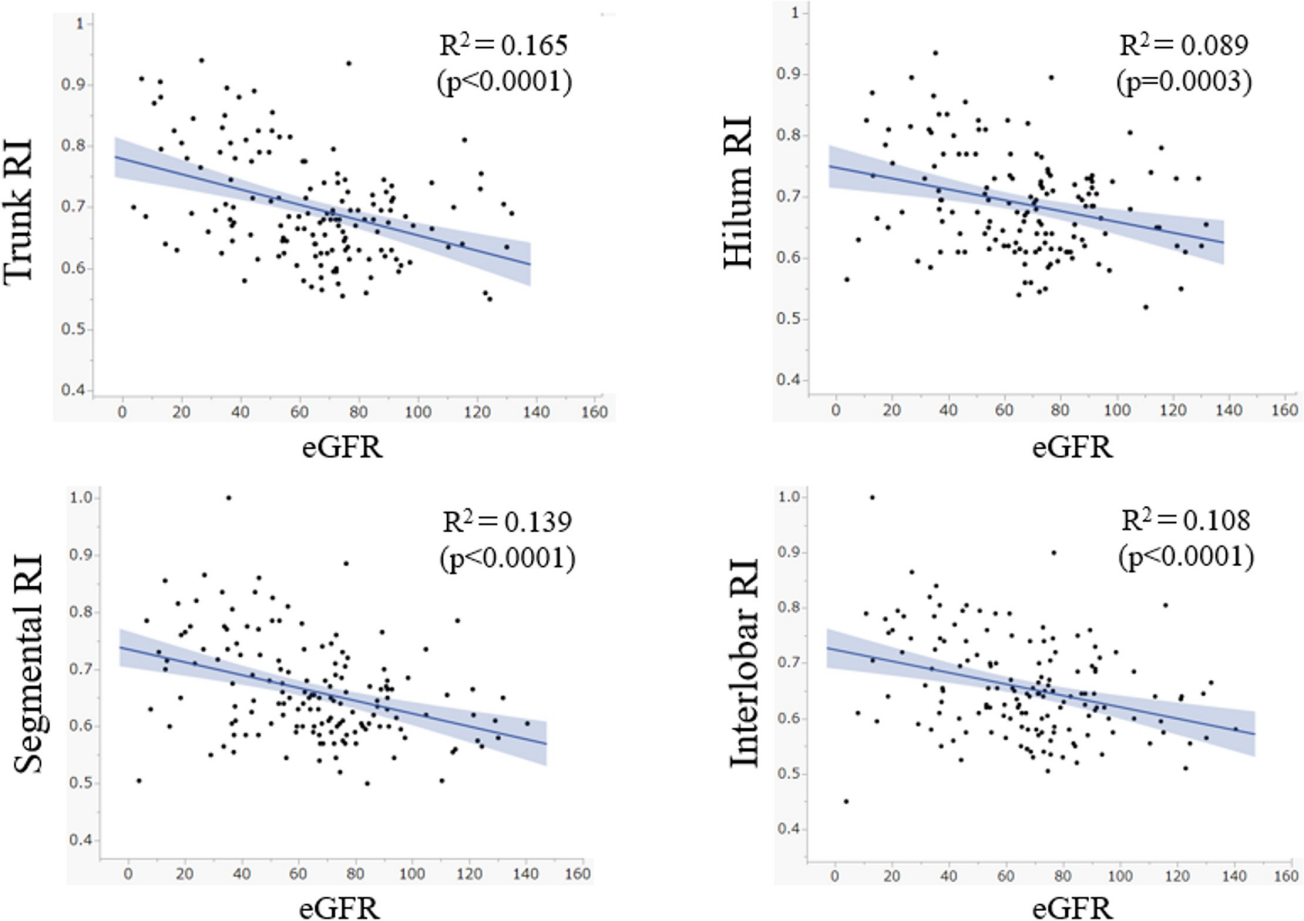
Scatter plots of eGFR and RI in each region with Doppler ultrasonography. Resistive indices in all 4 regions show significant negative correlations with eGFR. The grey shaded ranges show the 95% confidence interval of the regression lines.

The scatter plots of eGFR and RI for the 4 regions studied are shown in **Fig 4**. The values for all 4 regions showed weak to moderate correlation with eGFR. The correlation was stronger on the distal side (*i*.*e*. interlobar) and weaker on the proximal side (*i*.*e*. trunk). The strength of correlation was statistically similar between the distal side (interlobar) and the proximal side (trunk) (p = 0.430, z-test).

### Correlation between the ultrasonic and demographic data

**Fig 5:**
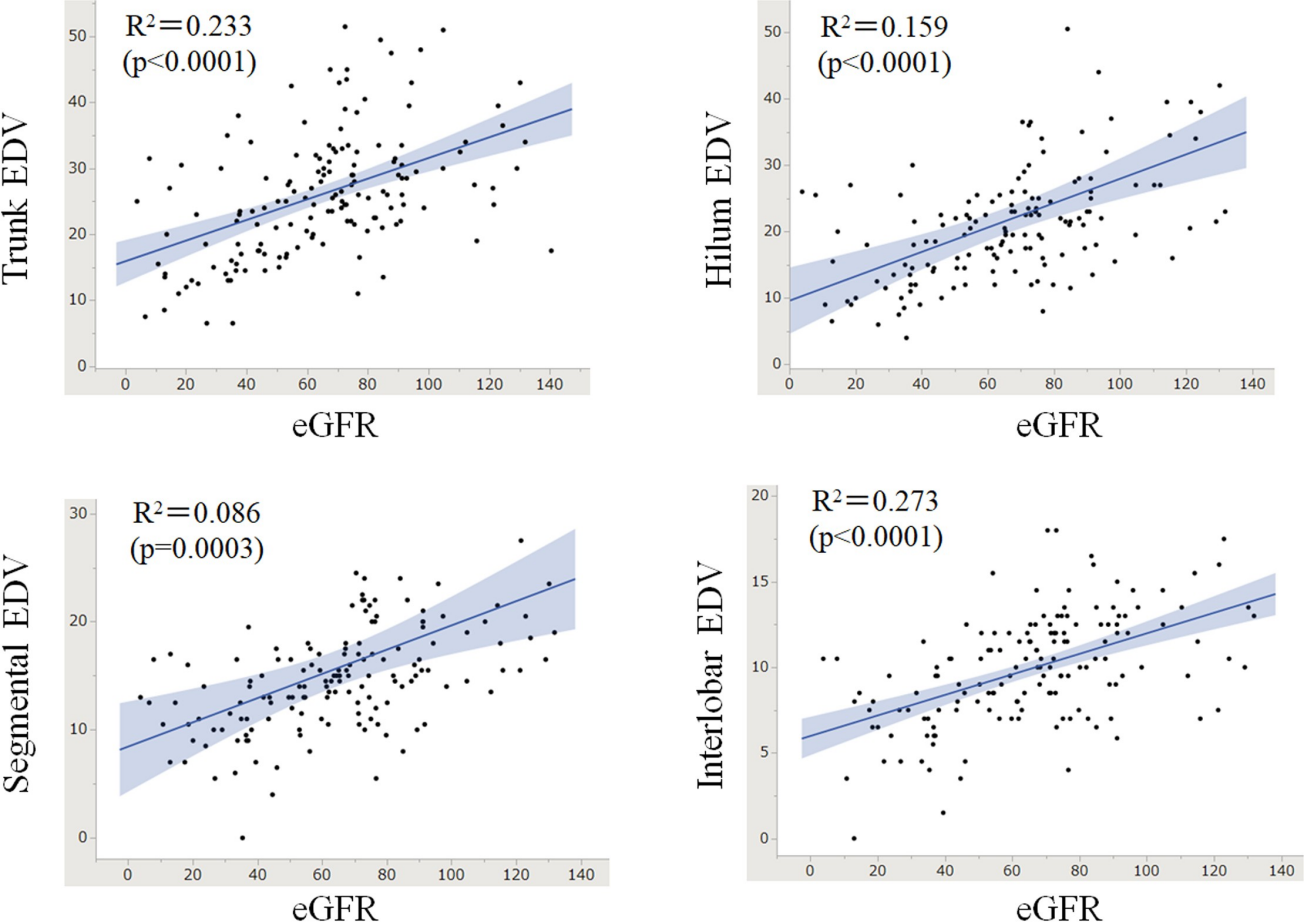
Scatter plots of eGFR and EDV in each region with Doppler ultrasonography. The EDV in all 4 regions show significant positive correlations with the eGFR. The grey shaded ranges show the 95% confidence interval of the regression lines.

The results of the Pearson’s R test between the ultrasonic and demographic data are listed in **Table 3**. Among the measured ultrasonic indices, EDV and RI showed moderate correlations with eGFR, but PSV showed a weak to moderate correlation with eGFR, suggesting that the EDV and the RI would be better markers to assess renal function than the PSV. The PSV in each region showed a stronger correlation with eGFR than with age. Additionally, RI showed a stronger correlation with age than with eGFR. The EDV showed almost similar correlations with age and eGFR. Considering the moderate correlations between EDV and eGFR, EDV would be the most appropriate ultrasonic index to evaluate renal function. For reference, the scatter plots between the EDV in each region and the eGFR are shown in **Fig 5**.

**Table 3:** Correlations between ultrasonic and demographic data.

		Age		eGFR	
		Pearson’s *R*	p	Pearson’s *R*	p
**Aortic PSV**		-0.483 (-0.355 - -0.594)	<0.0001	0.407 (0.268–0.530)	<0.0001
**Trunk**	**PSV**	-0.027 (-0.183–0.131)	0.739	0.225 (0.069–0.370)	0.0051
	**EDV**	-0.418 (-0.279 - -0.540)	<0.0001	0.483 (0.351–0.595)	<0.0001
	**RI**	0.463 (0.328–0.580)	<0.0001	-0.407 (-0.264 - -0.532)	<0.0001
**Hilum**	**PSV**	-0.309 (-0.154 - -0.449)	0.00014	0.465 (0.327–0.584)	<0.0001
	**EDV**	-0.444 (-0.304 - -0.566)	<0.0001	0.399 (0.252–0.527)	<0.0001
	**RI**	0.479 (0.343–0.479)	<0.0001	-0.298 (-0.142 - -0.439)	0.00028
**Segmental**	**PSV**	-0.036 (-0.193–0.123)	0.655	0.283 (0.130–0.423)	0.00039
	**EDV**	-0.319 (-0.168 - -0.454)	<0.0001	0.293 (0.140–0.432)	0.00025
	**RI**	0.590 (0.478–0.684)	<0.0001	-0.372 (-0.228 - -0.501)	<0.0001
**Interlobar**	**PSV**	0.022 (-0.137–0.179)	0.790	0.305 (0.153–0.442)	0.00013
	**EDV**	-0.473 (-0.340 - -0.587)	<0.0001	0.522 (0.396–0.629)	<0.0001
	**RI**	0.597 (0.485–0.689)	<0.0001	-0.327 (-0.178 - -0.462)	<0.0001

The ultrasonic data are averages from both sides of the kidneys. The bracketed ranges of Pearson’s *R* are the 95% confidence interval of the estimated correlation coefficients. Abbreviations: EDV, end-diastolic velocity; eGFR, estimated glomerular filtration rate; PSV, peak systolic velocity; RI, resistive index.

### Partial correlation between EDV and eGFR, adjusted by age

As described in **Table 3**, age and eGFR showed significant correlations with EDV in all 4 regions; in addition, age and eGFR showed a significant negative correlation. As a result, the partial correlation coefficients between the EDV in each region and the eGFR were examined by setting age as a covariate. The partial correlation coefficients with EDV in the trunk were -0.271 for age (p = 0.0007) and 0.367 for eGFR (p<0.0001); with EDV in the hilum were -0.350 for age (p<0.0001) and 0.277 for eGFR (p = 0.0008); with EDV in the segmental region were -0.228 for age (p = 0.0050) and 0.182 (p = 0.0250) for eGFR; and with EDV in the interlobar region were -0.345 for age (p<0.0001) and 0.405 for the eGFR (p<0.0001). In conclusion, both age and EDV were found to be independent factors for renal function, and EDV could be a reliable index to evaluate renal function.

### Partial correlation between RI and eGFR, adjusted by age

As described in **Table 3**, age and eGFR showed significant correlations with RI in all 4 regions. Thus, the partial correlation coefficient between RI in each region and each of the other variables was checked. The partial correlation coefficients with RI in the trunk were 0.359 for age (p<0.0001) and -0.276 for eGFR (p = 0.00064); with RI in the hilum were 0.429 for age (p<0.0001) and -0.112 for eGFR (p = 0.193); with RI in the segmental region were 0.528 for age (p<0.0001) and -0.107 (p = 0.196) for eGFR; and with RI in the interlobar region were 0.569 for age (p<0.0001) and -0.0432 for eGFR (p = 0.605). In conclusion, an RI would not be a reliable index to evaluate renal function because it is mainly affected by age rather than renal function.

### Comparison of EDV and RI as a marker of renal function with multivariate analysis

To compare the clinical importance of EDV and RI as physiological markers of renal function, a multiple linear regression analysis was performed using eGFR as the objective variable and age, interlobar EDV, and interlobar RI as the explanatory variables. The result of this multivariate regression analysis is shown in **Table 4**. As shown in the table, EDV was a much better marker than RI to estimate renal function. Similar results were also achieved in the other 3 regions studied.

**Table 4:** Multiple Linear regression analysis using eGFR as the objective variable.

	PRC (95% CI)	F value	SPRC	p value	VIF
**Age**	-0.435 (-0.742 - -0.129)	7.88	-0.245	0.0057	1.62
**Interlobar EDV**	4.04 (2.53–5.55)	27.85	0.454	< 0.0001	1.57
**Interlobar RI**	28.10 (-29.78–85.98)	0.92	0.090	0.34	1.88
**Constant**	31.14 (-13.36–75.64)	1.91	-	0.17	-

EDV may be a better physiological marker of renal function than RI. VIF is a marker of multicollinearity between the variable, values above 10 suggests the existence of problems based on multicollinearity. Abbreviations: EDV, end-diastolic velocity; PRC, partial regression coefficient; RI, resistive index; SPRC, standardized partial regression coefficient; VIF, variance inflation factor.

### Effects of BMI on the applicability of EDV

Next, we evaluated the possible effects of BMI on the clinical applicability of EDV as a physiological marker of renal function, because renal Doppler ultrasonography would be technically more challenging in obese patients. We separated the patients into those with BMI <25 (n = 112) and those with BMI ≥25 (n = 50). In the former group, the correlation coefficient between eGFR and EDV was 0.550 (95%CI: 0.398–0.673, p<0.0001); in the latter group, it was 0.461 (0.194–0.665, p = 0.0014). Although the correlation was slightly lower in obese subjects, the difference was not statistically significant (p = 0.49). Advanced technical skills are required to perform a renal ultrasonography in obese subjects; however, the results of this study show that renal ultrasonography is a useful method for the assessment of renal function even in obese subjects.

### Correlation of EDV with other blood and urine test data

The correlation coefficients between the interlobar EDV and the blood and urine test parameters other than eGFR were analyzed. The calculated Pearson’s correlation coefficient with the interlobar EDV for each laboratory parameter was: -0.011 for serum albumin-corrected calcium level (p = 0.92), -0.123 for serum inorganic phosphorus level (p = 0.23), 0.017 for serum magnesium level (p = 0.89), 0.037 for CRP (p = 0.73), -0.209 for HbA1c (p = 0.018), 0.053 for triglyceride level (p = 0.52), and 0.010 for total cholesterol level (p = 0.90). For reference, eGFR was not correlated with any of the laboratory markers.

## Discussion

Presently, renal arterial Doppler ultrasonography is performed mainly to diagnose renal arterial diseases, especially renal arterial stenosis [16,17]. Renal function tests for patients with CKD and blood tests (*i*.*e*. serum creatinine level, eGFR) have been widely performed for follow up until now. However, serum creatinine level is affected by a wide range of clinical factors, such as hemolysis, diet, muscle volume, and dehydration [18–21]. As the eGFR is estimated simply by age and the serum creatinine level in both the sexes, whether the serum creatinine level and the eGFR exactly reflect the glomerular function (*e*.*g*. filtration of uremic toxins) is unclear. It is unclear whether eGFR as estimated using creatinine level is more precise than that using cystatin C when measuring the true renal function. Compared to these blood tests, ultrasonic studies rapidly and directly reveal the condition of renal hemodynamics, such as blood flow and vascular resistance [22]. To understand the correct interpretation of data with renal Doppler ultrasonography for maximizing its clinical efficacy, comprehensive analyses with a sufficient number of subjects and a wide range of clinical variables are required.

In this study with comprehensive variables, including demographic data, blood test results, and ultrasonic data, EDV was found to be the most appropriate ultrasonic index to evaluate renal function. This is a novel insight for renal Doppler ultrasonography. Additionally, the value of RI, which was believed to be the best ultrasonic index to evaluate renal function, was found to be regulated mostly by age and that it is not a better marker of renal function than EDV. The PSV did not reflect renal function and would not be useful as a marker of renal function. This could be probably be because PSV is largely affected by extra-renal factors such as cardiac output, aortic stiffness, and renal arterial stenosis. In other words, an elevated PSV does not always suggest increased effective perfusion in the kidneys.

On analyzing the correlation between age, eGFR, and aortic PSV, a significant positive correlation was found between eGFR and aortic PSV, even after adjusting the effect of age. This suggests a possible association between elasticity of the aortic wall and a normal renal function. The aortic PSV has been known to reflect the elasticity of the aortic wall; a higher value of aortic PSV can theoretically reflect more elasticity [23,24]. Although the exact mechanism of the observed correlation between the aortic PSV and the eGFR is uncertain, better aortic blood flow would be important for maintaining the intrarenal EDV, which would be necessary to maintain renal function. However, as these 2 variables are affected by age, the aortic PSV would not be a better marker for renal function than intrarenal ultrasonic indices measured in more distal regions (*i*.*e*. trunk, hilum, segmental, interlobar).

This study has several limitations. First, the measured data with Doppler ultrasonography may differ among different examiners. To ensure that such inter-rater error did not affect the conclusions of this study, we checked the inter-rater variance of the ultrasonic data, which did not show any significant differences for the interlobar EDV and interlobar PSV between the examiners (p = 0.43 for interlobar EDV and p = 0.76 for interlobar PSV, ANOVA). This implies that the inter-rater error in renal Doppler ultrasonography could be minimized by well-trained ultrasonographers who understand the internationally standardized procedure. To minimize the risk of inter-facility error, it would be ideal to establish a normal range for each ultrasonic index according to age in each facility where Doppler ultrasonography is performed. Another limitation of this study was that the renal function was represented by eGFR, and renal pathological assessments with kidney biopsy or creatinine clearance test were not performed. Future investigations that evaluate the association between renal function and EDV are necessary to verify the conclusions of this study. Finally, as described in the results section, accurate measurement of ultrasonic indices could be relatively difficult and the values could be unreliable in obese subjects. We have to thus take extra care when interpreting ultrasonic data from obese subjects.

## Conclusions

Among the indices measured with renal pulsed-wave Doppler ultrasonography, the EDV, regardless of the regions evaluated, may be the best index to measure renal function. On the other hand, the RI, which is considered to be the best ultrasonic index to measure renal function, was strongly affected by age and may not be a better index than EDV. Assessment of the EDV is a novel, non-invasive, and reliable method for the long-term management of CKD patients.

## Supporting information

S1 Table Original dataset used in the article. Abbreviations: ACE/ARB, angiotensin-converting enzyme inhibitors/angiotensin receptor blockers; CCB, calcium channel blockers; EDV, end-diastolic velocity; PSV, peak-systolic velocity; RI, resistive index.
(XLSX)

## Abbreviations

CKD: chronic kidney disease
EDV: end-diastolic velocity
eGFR: estimated glomerular filtration rate
PSV: peak systolic velocity
RI: resistive index
BMI: body-mass index
DBP: diastolic blood pressure
SBP: systolic blood pressure
